# MPRO: A Professionalism Curriculum to Enhance the Professional Identity Formation of University Premedical Students

**DOI:** 10.1080/10872981.2021.1886224

**Published:** 2021-02-19

**Authors:** Gia Merlo, Hanjun Ryu, Toi B. Harris, John Coverdale

**Affiliations:** a Department of Psychiatry and Nursing, New York University, New York, NY, USA; b Office of Health Professions, Rice University, Houston, TX, USA; c Menninger Department of Psychiatry and Behavioral Sciences, Baylor College of Medicine, Houston, TX, USA

**Keywords:** Medical professionalism, premedical education, professional identity formation, reflection, physician shadowing

## Abstract

Limited opportunities exist for university premedical students to gain exposure to the realities of clinical practice through physician shadowing or through a formal curriculum. Medical Professionalism and Observership utilizes didactics, reflective writing, small- and large- group discussions, and clinical observerships to enhance the process of professional identity formation during a critical developmental window of late- adolescence. The pilot semester included a sample of 135 students, all in their sophomore, junior, or senior years of study at Rice University. Students were selected through an application process and paired with physicians at Houston Methodist Hospital based on specialty preference and availability. Students were required to participate in biweekly lectures and discussions and to submit a weekly reflection on topics discussed in the course and their shadowing experiences. Student evaluations were administered to survey changes in students’ knowledge and perceptions of the curriculum. Selected reflections were read for evidence of professional identity formation. Lectures increased students’ exposure to core competencies within the medical profession and influenced their desire to become physicians. Reflective writings demonstrated integration of these core competencies into the professional identity of students. Structured reflection and didactics, when coupled with physician shadowing, appear to promote integration of the values, beliefs, and attitudes of medical professionalism. Future studies should seek to demonstrate how such a curriculum affects professional identity formation through established measures, and to assess whether such a curriculum may influence students’ preparedness for medical training and practice as they progress along their careers.

## Introduction

Even prior to matriculation into medical school, exposure to curricula at the premedical level to foster professional identity development is likely to be valuable. Throughout medical training, there is an expectation that medical students will develop their professional identity by reflecting on and processing through concerns relating to ‘morality, conflicts of interest, the state of the doctor-patient relationship, self-regulation, and to the impact of the healthcare system on the practice of medicine’[1]. Though largely successful, this tradition within the medical profession has proven insufficient and, in many instances, unreliable in ensuring that physicians internalize the values, beliefs, and attitudes of the medical profession into their professional identity[2].

In recent history, a frequently referenced framework for medical professionalism has been behavior or outcome based, focusing on a set of core competencies that physicians should demonstrate[3]. These core competencies, which remained unwritten for centuries, were published by the Accreditation Council for Graduate Medical Education and American Board of Medical Specialties in 1999 and have guided a formal definition of medical professionalism[4]. According to this framework, medical professionals should exhibit certain behaviors, such as demonstrating compassion, integrity, and respect; being responsive to patient needs; and being accountable to patients, society, and the profession [5–8]. However, while a definition of medical professionalism has been provided, the development and uniform integration of a formal medical professionalism curriculum has been hindered by many factors, including differences in individual learning styles and developmental windows[9]. Due to a lack of formalized curricula, many medical students and trainees still depend on the ability to organically ‘catch-on’ to informal aspects of training without an inherent structure to support them. The knowledge gained through this process has been described by several authors as the hidden curriculum, which is likely to be insufficient for fully integrating the values of medical professionalism[10]. Consequently, this educational gap may lead to issues during medical practice, such as boundary violations[11], declines in empathy[12], physician burnout [13–15], and depression and suicide [16,17].

More recently, the Association of American Medical Colleges (AAMC) has recognized the importance of training physicians that demonstrate the values of medical professionalism. The AAMC has published a set of pre-professional competencies for entering medical students [18] and revised the structure of the Medical College Admission Test (MCAT) in order to reflect the changes in medicine that have highlighted the imperative for professionalism[19]. By encouraging academic medical centers to consider experiences and attributes not previously delineated as essential to practicing medicine, such as the value of interprofessional skills, cultural competence, and self-care, the AAMC has posited a holistic framework for recruiting and educating the next generation of physicians.

While there is no agreement on the optimal time for instilling the values of medical professionalism, adolescence to late-adolescence (12–20 years of age) is suggested to be a critical transition period in the process of identity formation from the stage of identity change to the stage of increased stability [20–22]. Consequently, as premedical education occurs during this period, we believe that investment in premedical students’ professional development and identity formation is likely to be worthwhile.

The Transformation in Medical Education (TIME) initiative established by the University of Texas System in 2012 proposed to emphasize professional identity formation at the premedical level[23]. The TIME initiative suggests a variety of activities for enhancing the process of professional identity formation, including formal courses, professional development workshops, reflective writing, and community service learning. However, to date, there are few examples in the literature of curricula that seek to support university premedical students in the early stages of professional identity formation. This is particularly significant in a pandemic and post-pandemic world, as premedical students are likely to be excluded from in-person clinical experiences, providing them less opportunity to be socialized within the practice of medicine.

Following a review of the literature, we identified 8 model programs supporting university premedical students pursuing a career in medicine (Table 1). [24–31] Though each of these programs aims to provide an opportunity for students to gain clinical exposure through physician shadowing, few integrate opportunities for reflection into the observership experience. For example, of these 8 programs, we found that only the Cornell University urban semester program, the Health Frontiers in Tijuana Undergraduate Internship Program at the University of California, San Diego, and the Stanford Immersion in Medicine Series incorporate opportunities for structured reflection. Even fewer programs provide a formal introduction to the principles of medical professionalism through formal didactics and group discussions. Of these 8 programs, the summer research, shadowing, and mentorship program at the University of South Florida and the Doctoring Undercover program at the University of Kentucky had a didactic component. The other programs, including the University of Wisconsin Tobacco Science Scholars Program, the shadowing program at Hendrix College, and the Educational Shadowing Program (ESP) at the University of California, Los Angeles did not have components of structured reflection or explicit didactics.

**Table 1.** Characteristics of model programs supporting premedical students in choosing medicine for a career

AUTHORS	n	Site/Setting	Length	Major Focus	Program Components	Requirements	Method(s) of Evaluation
**Hernandez et al. (2009)**	48	Division of Surgery, University of South Florida	Summer	Participation in scholarly activities	Faculty and resident led lectures, shadowing of surgeons, meetings with mentors, clinical research	Completion of one research project	Post survey, publications
**Davis et al. (2013)**	120 (over 2 years)	University of Wisconsin Tobacco Science Scholars	One semester	Clinical and research experiences	Clinical etiquette training, rounding with physicians and independent interactions with patients, research related to smoking cessation	Presentation of a patient and study data	Exit survey
**Goldstein et al. (2014)**	120 (over 2 years)	Cornell University	One semester	Physician shadowing	Physician shadowing, seminars, reflection journals on “cultural” differences between specialties	No formal requirements, participation in shadowing and critical reflection exercises	Exit survey
**Burgos et al. (2015)**	24	University of California, San Diego School of Medicine	Quarterly	Clinical experience, global health disparities	Preparatory training, on-site workshops, physician shadowing, journal club, group discussion	Completion of hours, reflection assignment	Completion of hours
**Penn (2015)**	106 (over 2 years)	Hendrix College	Variable	Physician shadowing	Preparatory training, attendance	Attendance	Individual comments
**Wang et al. (2015)**	61	Stanford University	4 months	Physician shadowing	Patient rights and professionalism training, and shadowing of a physician at least four times	Reflective essay	Pre- and post-surveys
**Clark (2016)**	20	Honors College, University of Kentucky	One semester	Teaching historical, social, and interpersonal contexts in contemporary healthcare	Course lectures, physician shadowing, reflective writings	Weekly blog posts	Weekly blog rubric with instructor feedback
**Thang et al. (2019)**	10 (in first year)	University of California, Los Angeles	One academic year (30 weeks)	Physician shadowing	Physician shadowing, assistance in administrative work	No formal requirements, participation in shadowing	Pre- and post-surveys

Cruess et al. have previously established the importance of ‘apprenticeship’ in teaching medical professionalism to medical students, residents and faculty[32]. This pedagogical model combines explicit didactics with experiential learning to aid in professional identity formation. Critically, the authors state that continual reflection throughout this process is essential for higher levels of growth and performance as medical professionals.

The objective of this study is to evaluate a premedical curriculum that aims to enhance the professional identity formation of learners. We seek to address identified gaps of other premedical programs by incorporating formal didactics, clinical observerships, and reflective writings to provide an opportunity for students to progress through the process of professional identity formation during a critical stage in their professional development.

## Methods

### The MPRO curriculum

MPRO is a focused curriculum offered each fall and spring semester for university premedical students in order to address the needs previously described. The program was developed following a needs assessment and a systematic literature review. The first author of the study then met in small groups with students and faculty to formalize a curriculum consisting of the aforementioned three elements: formal didactics, clinical observerships, and reflective writings. The entire curriculum, including all survey and evaluation instruments, received expedited review and was approved by Rice University’s Institutional Review Board (IRB) on 17 August 2015. All participants enrolled in this program provided informed consent for voluntary participation in the study and program elements involving data collection. MPRO couples physician shadowing at Houston Methodist Hospital with a 14 week program in medical professionalism at Rice University consisting of biweekly lectures and small- and large-group discussions, and weekly reflective writings.

MPRO utilizes a developmental approach to assist premedical students primarily with: (1) ascertaining if the profession is a ‘good fit’ (2) internalizing the core values of the medical profession (3) identifying coping strategies to improve well-being and promote resilience, and (4) identifying ways to mitigate potential lapses in professionalism.

### Student selection

Registration for this course is unique to other courses at the university. All students at the university are invited to apply for the course by email, and those who decide to apply are placed into pre-course registration. They are then required to complete an application, in which they provide a paragraph-length essay about their motivation for taking the class and their motivation for medicine. Students are also required to provide a brief CV. Applicants are evaluated for acceptance based on their breadth of previous experience, GPA, seniority, previous participation, availability, and completion of the hospital’s compliance requirements, which include a background check, drug screen, and submission of immunization records. Following this process, students receive a registered ID badge and are paired with physicians based on specialty preference and schedule compatibility.

### Observerships

The first author of the study worked with administrators at Houston Methodist Hospital to identify teaching physicians that were approved to have observers. These physicians were across multiple specialties, including both surgical and non-surgical specialties. Students were paired with physicians based on specialty interest. Based on the physician, students shadowed in different settings. Some students observed exclusively in surgery, some students observed exclusively in outpatient clinics or clinical rounds, and the majority of students observed across surgical and medical outpatient settings.

### Formal didactics

The sequencing of lectures was designed to complement and emphasize themes and concepts students may encounter during the clinical observership, which begins in the second half of the course. Students participate in eleven program lectures, including: (1) Teams in medicine; (2) Empathy, communication and interview skills; (3) Professional boundaries, social media and electronic health records; (4) Cultural competency; (5) Spirituality, humanism and aging; (6) Self-care, nutrition and happiness; (7) Substance use disorders; (8) Stress and burnout in physicians; (9) Physician depression, suicide, mental illness and difficult patients; (10) Personal financial considerations for physicians; (11) Ethical issues, end of life and genetics. Objectives for each of the lectures may be obtained upon request from the office. Each 75-minute lecture follows a multifaceted pedagogical approach, and consists of a presentation, case examples, videos, and small- and large-group discussions.

Post-test, to determine the impact of each individual lecture on student attitudes and desires to become a physician, we categorized student ratings of survey statements to the question, ‘Lecture X was relevant in influencing my desire to become a physician’ on a Likert scale from 1 (‘Strongly Disagree’) to 5 (‘Strongly Agree’).

### Reflective writing

The integration of a weekly reflective component is a unique element of the MPRO curriculum model. As described in the literature, ongoing reflection is necessary for professional identity formation [33,34]. In MPRO, a reflective component is included in order to support the internalization of values and beliefs that students may encounter in class or in the clinic during their observerships.

Gibbs’ Reflective Cycle was chosen as a theoretical framework for reflective writing for its ease of use as a systematic reflective method for experiential learning. Gibbs’ Reflective Cycle consists of 5 discrete components- description, feeling, evaluation, conclusion and action- through which reflectors sequentially move through in order to learn from an experience. In the first half of the course, students are encouraged to reflect on themes discussed in the didactic components of the class, such as the importance of cultural competence in end-of-life care or how social media has blurred professional boundaries. During the second half of the course, students are prompted to integrate what they have learned in class with what they observe in clinical practice through reflection.

In order to illustrate the potential value of the curriculum in enhancing professional identity formation, we selected comments from student reflections and identified instances of questioning and reevaluating what it means to be a physician, internalization of goals and aspirations as future medical professionals, and evidence of being socialized within the practice of medicine.

## Results

138 premedical students enrolled in MPRO from August to December 2015, and 135 students completed the pilot program. Three students dropped the course before it began. All participants were in their sophomore (n = 15), junior (n = 51), or senior year (n = 63) at Rice University or preferred not to provide their year of study (n = 6). At the end of the course, 6 students stated that their desire to become a physician had decreased, 123 students stated that their desire had remained the same, and 6 students stated that their desire had increased. The influence of individual lectures on student attitudes is presented in Table 2.

**Table 2.** Influence of lectures on desire to become a physician: MPRO didactic survey outcomes (August – December 2015)

MPRO Lecture	Lecture Influence (mean)	Standard Deviation
(1) Teams in Medicine	3.59	0.9
(2) Empathy, communication, and interview skills	3.89	0.89
(3) Professional boundaries, social media, and electronic health records	3.39	0.95
(4) Cultural competency	3.94	0.94
(5) Spirituality, humanism, and aging	3.78	1.05
(6) Self-care, nutrition, and happiness	3.93	0.88
(7) Substance use disorders	2.71	1.01
(8) Stress and burnout in physicians	3.94	0.84
(9) Physician depression, suicide, mental illness, and difficult people	3.75	0.98
(10) Personal financial considerations for physicians	3.87	0.88
(11) Ethical issues, end of life, and genetics	3.78	1.03

Lecture influence was determined by students’ response the survey question, ‘This lecture was relevant in influencing my desire to become a physician,’ which was administered post-test. Responses were categorized on a 5-point Likert scale from 1 (Strongly Disagree) to 5 (Strongly Agree).

### Student reflective writings

Students were required to reflect on their experiences throughout the course. We selected some comments to illustrate the potential value of the curriculum. Many students stated that before taking the course, they had little understanding of the daily activities of physicians and the challenges that they face.

One student stated, ‘Prior to [MPRO], my knowledge of medical professionalism was very limited. I focused on the technical aspects of what it means to be a physician, the tangible and visible aspects such as diseases and treatments. My motivations mainly centered on the idea that as a physician, I would strive to provide the utmost care and support for individuals who need it. However, throughout this course, I’ve realized that being a physician first requires that I am cognizant of my emotions, my thoughts, my biases, and my assumptions before I can work to improve the lives of others.’

Many students stated that the course had helped them to recognize the complexities of being a physician. The same student stated, ‘I’ve learned that being a physician requires so much more than simply recognizing diseases and treatments. I must learn to be aware of my biases and assumptions that may impact patient care. I must develop ethical values that will not be swayed by stress or pressure. I must prioritize communication and empathy so that I am able to establish meaningful relationships with my teammates and my patients. I must learn to value my own health, through mindfulness, nutrition, and exercise, before I focus on the health of others.’

Another student stated, ‘My understanding of what being a doctor entails has developed tremendously throughout this course. Learning about current issues that physicians face today opened my eyes to several aspects of the profession that I had not considered. The lecture that I remember striking me the most early on was the lecture about physician burnout. When I heard that about 50% of physicians experience symptoms of burnout, I was saddened and shocked. I did not know that the profession has become that stressful. While this shocked me at first, it only proved to be helpful for me to know because along with this lecture, we learned about how to cope with stress. I now know that I should be working on coping skills early on so that I can be prepared for the obstacles I face in the future.’

Similarly, another student reflected on the future of healthcare and the changing role of physicians due to artificial intelligence, stating, ‘The most insightful and perhaps earth-shattering topic for me was probably the future of technology in medicine. I’ve come to terms with the looming technology takeover of medicine and I’m challenging myself to consider medicine from a broader perspective. Artificial intelligence and machine learning threaten to present the ultimate challenge to the humanity of the doctor-patient relationship. What can I, as a future doctor, do to prevent technology from further dehumanizing the patient?’

## Discussion

The implementation of the MPRO curriculum demonstrates that a curriculum that aims to enhance professional identity formation can be implemented in a university premedical setting. The MPRO curriculum was one of the first, to the best of our knowledge, to combine formal didactics, physician shadowing, and structured reflection. While lectures may increase students’ understanding of the core competencies and expectations of the medical profession, ongoing reflection may allow students to better integrate these values, beliefs, and attitudes into their professional identity.

Students generally found the content and discussion of the didactics portion of the program to be both helpful and insightful. Participating in an integrated curriculum with reflective assignments bridging the didactics to the observership components appears to facilitate critical understanding and integration of the components of medical professionalism. Interestingly, while our results suggest that this course had little overall effect in changing students’ desires to pursue careers in medicine, our results also suggest that many of the individual lectures had substantial influence.

This curriculum may have particular value for premedical students during the COVID-19 pandemic, as students face even more limited opportunities to gain exposure to the realities of clinical practice[35]. Due to social distancing requirements, premedical students have been largely excluded from in-person experiences, and the American Medical Association has stated that they are unaware of how long these limitations will last[36].

While physician shadowing may currently be on hold in many premedical programs, a course consisting of didactics and a structured reflection component may nevertheless allow premedical students to develop a better understanding of the field of medicine, and allow them to progress along the vital process of professional identity formation. Because the lectures include case studies and clinical vignettes, students will be exposed to clinical information they may have encountered in their observerships. We also believe that small- and large-group discussions, as well as structured reflection, should help to reinforce the internalization of the values of the profession. Additionally, we believe that the MPRO curriculum is fully adaptable to an exclusively tele- form. The first author is currently adapting this curriculum at another university using only tele- forms of dissemination and shadowing. By using audiovisual recording devices, students may be able to appropriately shadow physicians while conforming to social distancing requirements.

This study has several limitations. The study was conducted at a small private research university, and this pilot study had a limited sample size of 135 students. The study population may not accurately reflect the premedical student population at other institutions, particularly because the course consisted of sophomores, juniors, and seniors who have already expressed an interest in medicine, many of whom may already have applied or be committed to applying to medical school. There is also potential selection bias that arises as a result of an application process that seeks to identify highly motivated students who generally aspire to high standards of professionalism. Future research studying cohorts that are less certain about their desire to be a physician, such as first-year students, might have value in assessing the effectiveness of this curriculum. Future studies should seek to demonstrate how this curriculum affects the professional identity formation of students through established measures such as the professional identity essay (PIE) and the Defining Issues Test (DIT2)[37]. Future studies should also seek to assess why certain lectures had greater influence on students than others. Finally, a longitudinal study that examines students’ preparedness for medical training and practice as they progress along their careers may also be valuable for assessing the effectiveness of this curriculum.

Our study shows that integrating formal didactics, physician shadowing opportunities, and reflective writings may serve to promote professional identity formation during a critical developmental period. The results of our evaluation of the MPRO curriculum suggest that the combination of medical professionalism and a physician observership program may be valuable for providing university premedical students greater insight into what it means to be a medical professional. This curriculum continues to be provided each semester at the same university. However, based on student feedback and annual reviews of the literature, MPRO has undergone several revisions, each time modifying the course sequencing, class size and didactic components to meet the needs of a diverse group of students. Following the pilot group, MPRO reduced the number of students enrolled to offer more individualized feedback for participants. Our hope is that the MPRO curriculum may serve as a guide for developing curricula that support university premedical students in integrating the values, attitudes, and beliefs of the medical profession into their identity.
